# CIP2A is an Oct4 target gene involved in head and neck squamous cell cancer oncogenicity and radioresistance

**DOI:** 10.18632/oncotarget.2670

**Published:** 2014-12-05

**Authors:** Sami Ventelä, Eleonora Sittig, Leni Mannermaa, Juho-Antti Mäkelä, Jarmo Kulmala, Eliisa Löyttyniemi, Leena Strauss, Olli Cárpen, Jorma Toppari, Reidar Grénman, Jukka Westermarck

**Affiliations:** ^1^ Turku Centre for Biotechnology, University of Turku and Åbo Akademi, Turku, Finland; ^2^ Department of Physiology, University of Turku, Finland; ^3^ Institute of Biomedicine and Turku Center for Disease Modeling, University of Turku, Finland; ^4^ Department of Pathology, University of Turku, Finland; ^5^ Department of Biostatistics, University of Turku, Finland; ^6^ Department of Otorhinolaryngology – Head and Neck Surgery, Turku University Hospital, Turku, Finland; ^7^ Department of Pediatrics, Turku University Hospital, Turku, Finland; ^8^ Department of Oncology and Radiotherapy, Turku University Hospital, Turku, Finland

**Keywords:** Serine 62 phosphorylated MYC, PLZF, Nanog, spermatogonia, PP2A, tissue microarray, CD44+, CD24+

## Abstract

Radiotherapy is a mainstay for treatment of many human cancer types, including head and neck squamous cell carcinoma (HNSCC). Thereby, it is clinically very relevant to understand the mechanisms determining radioresistance. Here, we identify CIP2A as an Oct4 target gene and provide evidence that they co-operate in radioresistance. Oct4 positively regulates CIP2A expression both in testicular cancer cell lines as well as in embryonic stem cells. To expand the relevance of these findings we show that Oct4 and CIP2A are co-expressed in CD24 positive side-population of patient-derived HNSCC cell lines. Most importantly, all Oct4 positive HNSCC patient samples were CIP2A positive and this double positivity was linked to poor differentiation level, and predicted for decreased patient survival among radiotherapy treated HNSCC patients. Oct4 and CIP2A expression was also linked with increased aggressiveness and radioresistancy in HNSCC cell lines. Together we demonstrate that CIP2A is a novel Oct4 target gene in stem cells and in human cancer cell lines. Clinically these results suggest that diagnostic evaluation of HNSCC tumors for Oct4 or Oct4/CIP2A positivity might help to predict HNSCC tumor radioresistancy. These results also identify both Oct4 and CIP2A as potential targets for radiosensitation.

## INTRODUCTION

Head and neck cancer is the 6^th^ most common cancer worldwide and 90% of these cancers are diagnosed as HNSCCs (head and neck squamous cell carcinoma, [1]). Surgery is the traditional and sufficient treatment of small and local HNSCC tumors. In order to achieve a better locoregional control in advanced HNSCC tumors, surgery is generally combined with chemoradiotherapy. However, it is generally acknowledged that one of the major barriers for successful HNSCC treatment is high radioresistance of HNSCC cells. Therefore, despite of recent advancements in adjuvant therapies and imaging modalities, the overall prognosis of advanced HNSCC has not improved significantly; the five-year overall survival among these patients is approximately 50% [2]. Recently, many different approaches have been introduced to clarify the cause of HNSCC aggressiveness and poor patient survival. These include mutation analyses [3–5], locoregional diversity of HNSCC cancers [6] and mechanisms of radio/chemosensitivity [7, 8]. Moreover, features linked to cancer stem cells, such as self-renewal and pluripotency have also been hypothesized to be one explanation for the aggressiveness and therapy resistancy of HNSCCs [9, 10]. Despite these activities, the mechanisms behind recurrency of HNSCC cancers after adjuvant therapies are still poorly understood. This has prevented development of diagnostic methods for stratification of patients having more aggressive subtypes of HNSCCs for more aggressive therapies and thus potentially better clinical outcome. Also, identification of mechanisms behind radioresistancy of HNSCC cells might provide novel opportunities for radiosensitation of HNSCC cells.

Octamer-binding transcription factor 4 (Oct4) is highly expressed in embryonic germ, stem, and testicular cancer cells [11, 12]. In embryonic stem (ES) cells Oct4 has a critical function in self-renewal and differentiation by regulating the pluripotent potential of these cells [13, 14]. Recent studies have suggested that Oct4 is also expressed in many other tumours than those of embryonal or testicular origin, such as HNSCC, breast and lung cancers [15–18]. Moreover in recent studies, increased Oct4 expression in cancers has been linked to cancer stem cell phenotype [19], radioresistancy [20] and poor prognosis of cancer patients [21, 22] although Oct4 link to cancer aggressiveness seems to be somewhat controversial [23]. Many known Oct4 target genes are directly involved in stemness regulation of normal and cancerous cells, but we know much less about potential targets of Oct4 that could regulate other characteristics of aggressive growth such as proliferation, apoptosis resistance or senescence evasion. Moreover, to date, the target genes regulated by Oct4 in HNSCCs are not known.

Cancerous Inhibitor of Protein Phosphatase 2A (CIP2A) is an oncogene that inhibits the tumour suppressor PP2A in many different cancers, including HNSCCs [24–26]. Several recent studies have shown that increased CIP2A promotes malignant cell growth, *in vivo* tumour formation [25, 26]. Clinically, high CIP2A expression correlates with worsened patient survival in more than dozen different cancer types [25]. Linked to its function as an inhibitor of PP2A, a master regulator of cellular signaling, CIP2A expression promotes various cancer driver pathways and thus many aspects of aggressive cell growth such as proliferation, apoptosis resistance or senescence evasion [27, 28]. Importantly, CIP2A is expressed at very low level in other normal tissues than testis, and its systematic inhibition do not cause detrimental consequences to normal mouse development and viability [28, 29]. However, CIP2A-deficient mice do show decreased Her2-driven mammary tumor development [28]. Therefore inhibition of CIP2A may have clinical relevance in development of future cancer therapies. In our recent work we demonstrated that CIP2A is highly expressed in testicular stem cells and has a role in regulation of spermatogonial progenitor proliferation. Moreover, spermatogonial cells isolated from CIP2A mutant mice showed reduced expression of Plzf (promyelocytic leukaemia zinc finger) and other stem cell renewal-associated genes, suggesting a role for CIP2A in testicular stem and progenitor cells. However, the functional relationship between CIP2A and stem cell renewal genes, such as Oct4 is not clear. Also, the potential role for CIP2A in mediating radioresistancy of HNSCCs has not been addressed thus far.

In this work we identify a novel function for stem cell regulator Oct4 in regulating oncoprotein CIP2A expression. Functionally, we demonstrate that Oct4/CIP2A double positivity is associated with radioresistancy in both normal spermatogonial cells, as well as in HNSCC. Clinically these results suggest that diagnostic evaluation of HNSCC tumors for Oct4 or Oct4/CIP2A positivity might help to predict HNSCC tumor radioresistancy.

## RESULTS

### Oct4 and CIP2A are expressed in radioresistant cell population in the mouse testis

**Figure 1:**
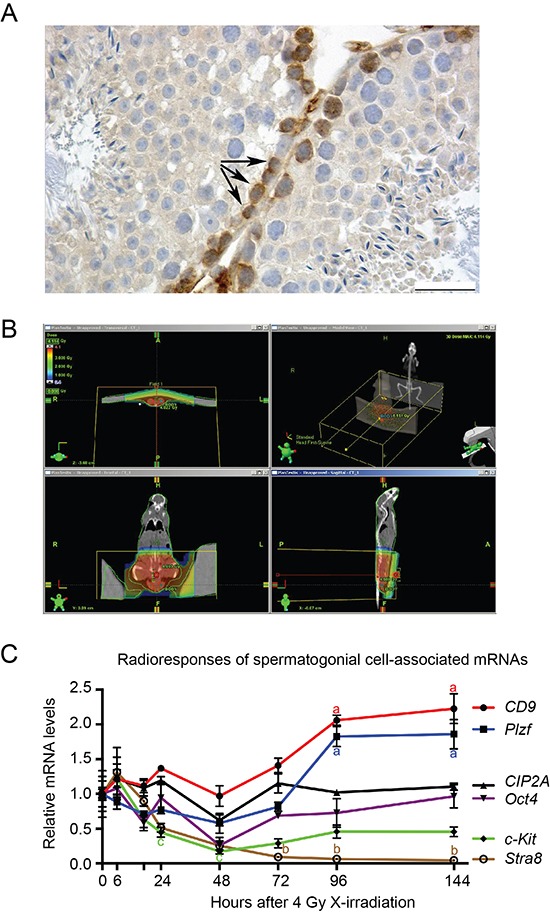
Oct4 and CIP2A are expressed in radioresistant spermatogonial stem cell population **(A)** Immunohistochemical staining of CIP2A in the adult mouse testis show highest CIP2A-positivity in spermatogonial cells locating most basally in seminiferous tubules (arrows). The bar represents 25 μm. **(B)** Representative CT scan image from mouse and tissue specific X-irradiation scattering. Radiation distribution in a mouse can be seen in colours and in axial and AP directions. Testes are contoured with red lines, where radiation dose is 4 Gy. **(C)** Expression of spermatogonial cell-associated markers in adult mouse testis 0–144 hours after X-irradiation. Steady state levels of CD9 and Plzf mRNA were elevated by the treatment, whereas c-Kit and Stra8 levels were reduced. CIP2A and Oct4 levels were relatively stable and closely mimicked each other's pattern of expression. GOI, gene of interest; *n* = 3–7, SEM; a, *p* < 0.001; b, *p* < 0.05 when compared to 0 h (= control) value; c, *p* < 0.05 when compared to 6 hours after X-irradiation. Statistical significancies were tested using one-way ANOVA and Dunnett post hoc tests. Letters a, b and c next to the error bars are in different colors based on the color of the line marking the gene.

Previous studies have demonstrated that testicular stem cells (spermatogonia) contain great pluripotent capacity and mimic in many ways embryonic stem cells [30, 31]. CIP2A is expressed in testicular stem cell/progenitor population (Fig. 1A) and our recent results suggest that CIP2A promotes self-renewal of normal testicular spermatogonia expressing *Oct4* and *Plzf* [29]. To study whether CIP2A is expressed in the radioresistant stem cell population, we used a novel approach to identify the spermatogonial genes involved in stemness based on their expression profiles in response to *in vivo* irradiation [32]. To avoid systemic side-effects, mouse testes were X-irradiated with 4 Gy under CT-scan guidance (Fig. 1B; [32]). Changes in gene expression profiles in response to *in vivo* irradiation were studied as a function of time. Spermatogonial genes that did not show inhibition of expression were considered to be expressed in radioresistant spermatogonial stem cells [32]. Expression of stra8 and *c-Kit*, which are markers of more differentiated spermatogonia [33–35], expectedly collapsed in response to 4Gy X-irradiation (Fig. 1C). However, expression of *Oct4* or CIP2A did not significantly change over the 144-hour observation period (Fig. 1C), whereas the spermatogonial markers *Plzf* and *CD9* showed a strong increase at 96 and 144 hours after irradiation, coinciding with increased proliferation and repopulation of the spermatogonia. Regarding CIP2A and Stra8 these results were confirmed by immunohistochemical staining of testis samples 144 hours after irradiation (Supplementary Figure 1). These results indicate that expression of both CIP2A and Oct4 is linked to cellular radioresistance *in vivo*.

### CIP2A is an Oct4 target gene in testicular cancer cells and in embryonic stem cells

**Figure 2:**
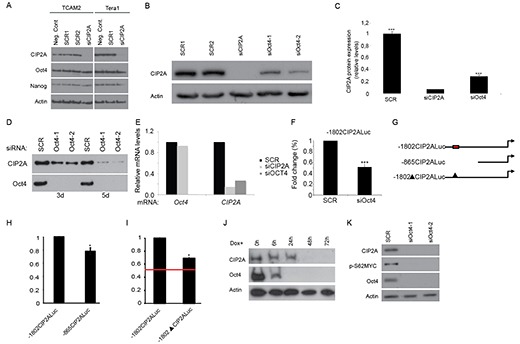
Oct4 regulates CIP2A expression **(A)** Western blot analysis of CIP2A, Oct4 and Nanog expression levels from two different testicular cancer cell lines (Tcam2 and Tera 1) 72 hr after transfection with normal medium (negative control), scrambled siRNA (Scr) and CIP2A siRNA (siCIP2A). Actin was used as a loading control. **(B)** Western blot analyses of CIP2A expression after transfection with scrambled siRNA (Scr), CIP2A siRNA (siCIP2A) and two different Oct4 siRNAs (siOct4-1, siOct4-2) from Tcam2. **(C)** Quantitation of CIP2A protein levels from three independent siCIP2A and siOct4 experiments identical to shown in B. Shown is mean +SD of three experiments. ***p < 0.001. **(D)** Time course analysis of CIP2A expression in Oct4 siRNA transfected cells. **(E)** *Oct4* and *CIP2A* qRT-PCR analyze in Tcam2 cell line after 5 days treatment with Oct4 or CIP2A siRNA. **(F)** Regulation of CIP2A promoter activity by Oct4. Tcam2 cells transiently transfected with CIP2A promoter/luciferase constructs were transfected with Oct4 siRNA and the relative promoter activity was analysed after 72 hours. Shown is mean +SD of 3 experiments. ***p < 0.001. **(G)** Schematic figure of CIP2A promoter constructs and putative Oct4 binding region. Red box indicates Oct4 binding region in CIP2A promoter, which is absent from both -865CIP2Aluc and -1802ΔCIP2Aluc constructs. **(H)** -865CIP2Aluc fragment significantly decreased promoter activity in Tcam2 cells. Shown is mean ± SEM of 3 experiments. *p < 0.05. **(I)** Deletion of putative Oct4 binding region decreased promoter activity of -1802CIP2Aluc significantly in Tcam2 cells, but the inhibition did not fully reach the level of inhibition observed by Oct4 RNAi (red line). Shown is mean ± SEM of 3 experiments. *p < 0.05. **(J)** Western blot analyses of CIP2A and Oct4 expression in mESC model where Oct4 downregulation and ES cell differentiation is achieved after doxycycline addition (Zhbtc4f). **(K)** Protein expression of CIP2A, Oct4 and p-S62MYC from Tcam2 cells 72 hr after transfection with scrambled or two different Oct4 siRNA.

Results above, together with our previous results [29] indicate that CIP2A expression is linked to expression of Oct4 in normal mammalian progenitor cells. However, these studies have not yet addressed whether these genes regulate each other's expression. Testicular cancer (TC) is a good model to study regulatory mechanisms related to cell stemness [36]. To further study the possible link between CIP2A and Oct4 we used two different TC cell lines derived from either seminoma (Tcam2) or embryonal carcinoma (Tera1). When CIP2A siRNA was used in Tcam2 and Tera1 cells, an effective downregulation in CIP2A protein levels was detected, whereas neither Oct4 nor Nanog levels were affected (Fig. 2A). Similar results were seen when ES cells originating from CIP2A hypomorphic blastocyst were studied. Even though *CIP2A* expression levels in CIP2A hypomorphic ES cells were below the level of detection, *Nanog* and *Oct4* levels were not significantly decreased (Supplementary Fig. 2). These data suggest that CIP2A is not an upstream regulator of Oct4.

To test whether Oct4 instead regulate CIP2A, we performed parallel transfection of siCIP2A, and siOct4 in Tcam2 cell line. Importantly, two independent Oct4 siRNAs were found to potently inhibit CIP2A protein expression (Fig. 2B, C). Suggestive of functional relevance, the downregulation of CIP2A expression by Oct4 RNAi was even further pronounced after 5 days (Fig. 2D). To study the molecular mechanism by which Oct4 regulates CIP2A expression, we examined CIP2A mRNA regulation in cells transfected with either Oct4 or CIP2A siRNA. Both siRNAs potently inhibited CIP2A mRNA expression (Fig. 2E), and as was observed at the protein level, CIP2A inhibition did not affect Oct4 expression (Fig. 2E). Furthermore, consistent with function of Oct4 as a transcription factor, a luciferase promoter assay, using previously characterized −1802 bp fragment of CIP2A promoter [37], demonstrated that Oct4 regulates CIP2A expression at the promoter level (Fig. 2F). Importantly, bioinformatics analysis of −1802 bp promoter fragment identified putative octamer binding elements at region −1650 to −1600 (Fig. 2G red box, and Supplementary Fig. 3). To map whether these sites could mediate CIP2A promoter activity in Oct4 positive Tcam2 cells, we used a shorter CIP2A promoter fragment in which the putative Oct4 binding region is absent (Fig. 2G). Importantly, as compared to 1802 bp promoter fragment, this shorter 865 bp fragment displayed significantly decreased promoter activity (*p* = 0.021; Fig. 2H). Interestingly, previous study using human gastric cancer cells demonstrated that the 865 bp promoter fragment had even increased activity as compared to 1802 bp fragment [37], suggesting that the observed effect might be related to stem-like characteristics of Tcam2 cells. To directly assess the contribution of putative Oct4 binding sites in the context of 1802 fragment, we cloned a mutant version of the −1802CIP2ALuc in which the region −1650 to −1600 was deleted (Fig. 2G, −1802ΔCIP2ALuc). As shown in figure 2I, deletion of putative Oct4 binding sites decreased the promoter activity of −1802CIP2ALuc significantly (*p* = 0.021). However, the inhibition did not fully reach the level of inhibition observed by Oct4 RNAi (Fig. 2I red line), indicating that additional Oct4 binding sites, or sites through which Oct4 increases CIP2A transcription co-operatively with other transcription factors, may exist downstream of the identified region mutated here.

Results above identify CIP2A as a novel Oct4 target gene in testicular cancer cells. To clarify whether similar regulatory pathway between Oct4 and CIP2A exists also in embryonic stem cells, we adopted a widely used murine ES cell model (Zhbtc4; [32, 38]) in which Oct4 can be conditionally downregulated by doxycycline treatment. Similarly to TC cell lines, in mES cells Oct4 downregulation is accompanied by a decrease in CIP2A levels (Fig. 2J). Increased phosphorylation of transcription factor MYC at serine 62 is a hallmark of CIP2A function in cancer cells [25]. To test whether Oct4 depletion resulted in functional outcome of CIP2A inhibition, we studied pS62MYC expression after Oct4 siRNA treatment. Importantly, Oct4 depletion resulted in potent inhibition of expression of both CIP2A and pS62MYC (Fig. 2K). Consistent with the post-translation mechanism by which CIP2A regulates MYC phosphorylation [39], and with previously published results [24, 40], no effects on *c-myc* mRNA expression was observed by Oct4 siRNA (data not shown).

**Figure 3:**
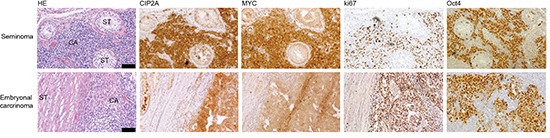
CIP2A is co-expressed with MYC, ki67 and Oct4 in testicular cancers Representative images after CIP2A, MYC, ki67 and Oct4 immunohistochemical staining in two different human testicular cancer samples, seminoma and embryonal carcinoma. Black bar represents 100 μm.

To study whether Oct4 and CIP2A are co-expressed *in vivo* in a cancer type that is characterized by stem cell–like cell growth, twenty TC patient samples were subjected to immunohistochemical staining with specific CIP2A and Oct4 antibodies [24, 41]. In addition, MYC and ki67 expression were also analysed. All studied testicular cancers were positive for Oct4 and ki67 (Fig. 3 and Table 1). Only 1/20 of Oct4 and ki67 positive TC samples did not show co-expression with CIP2A (Fig. 3 and Table 1). Oct4 and CIP2A co-expression with MYC in TCs was also very obvious and only two cancer samples were MYC negative (Table 1).

**Table 1:** Immunopositivity of CIP2A, MYC, ki67 and Oct4 expression among testicular cancer patient samples

	CIP2A	MYC	ki67	Oct4
*n*	20	20	20	20
Positive	19/20	18/20	20/20	20/20
%	95	90	100	100

Together these results demonstrate that CIP2A is a novel Oct4 target gene in normal and malignant stem cell-like cells, and that they are co-expressed *in vivo* in testicular cancers with characteristics of stem cell–like cell growth. As their regulation is unidirectional, and CIP2A does not regulate either Oct4 or Nanog, it is likely that CIP2A does not regulate stemness of TC cells. Instead CIP2A regulation by Oct4 may expand Oct4 functions to regulation of CIP2A-dependent processes such as regulation of oncogenic MYC phosphorylation, proliferation and apoptosis resistance.

### CIP2A and Oct4 are co-expressed in HNSCC cell lines

**Figure 4:**
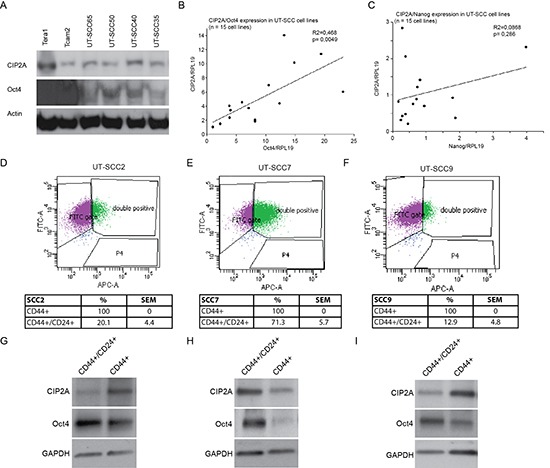
CIP2A and Oct4 are co-expressed in HNSCC cell lines **(A)** Western blot analyses of CIP2A and Oct4 expression in testicular cancer (Tera1, Tcam2) and four different HNSCC cell lines (UT-SCC). **(B-C)** Relative mRNA expression levels of CIP2A, Oct4 and Nanog were studied with quantitative PCR (qPCR) from 15 different HNSCC cells lines (UT-SCC). RPL19 (ribosomal protein L19) was used as an endogenous control gene to normalize expression levels of CIP2A, Oct4 and Nanog before linear regression analysis. **(D-F)** Representative dot plot figures from UT-SCC2, -7 and -9 cell line cell sorting. Percentages for CD44+ and CD44+/CD24+ side populations are means of three independent experiments per cell line. **(G-I)** Representative figures of western blot analysis of CIP2A and Oct4 expression levels in sorted CD44+ and CD44+/CD24+ UT-SCC2, -7 and -9 cell populations. GAPDH was used as a loading control.

To study whether relationship between Oct4 and CIP2A also exists in other cancers than TCs, we set to study patient derived HNSCC cell lines for CIP2A and Oct4 protein and mRNA expression. HNSCC was selected based on previous validation of CIP2A as an HNSCC oncoprotein [24, 42], and suggested importance of Oct4 and other stem cell regulators in the therapy resistance of HNSCCs [22, 43, 44]. Notably, all studied patient-derived HNSCC cell lines expressed CIP2A and Oct4 proteins (Fig. 4A). However, as compared to TC cell lines (Tera1, Tcam2), Oct4 expression levels were clearly lower in HNSCC cell lines, which is in line with the true stem cell characteristics of TCs. Next we recorded the mRNA expression levels of CIP2A and Oct4 in 15 different patient-derived HNSCC cell lines. In this analysis, statistically significant co-expression correlation existed between *CIP2A* and *Oct4* expression (*p* = 0.0049; Fig. 4B). Importantly correlation between *CIP2A* and *Oct4* expression was specific to this gene pair as no correlation was observed between *CIP2A* and *Nanog* expression (*p* = 0.286; Fig. 4C).

Oct4 is expressed in several cancer types and its expression has been linked with increased cancer cell stemness and tumorigenicity [12, 17, 21]. Role of CIP2A in promoting expression of stemness markers and proliferation of spermatogonial progenitor cells [29], together with our data that CIP2A is a novel Oct4 regulated gene (Fig. 2), suggests that CIP2A might also be expressed in cancer stem cell like cells, however this has not been studied as yet. To study the nature of cell population in which Oct4 and CIP2A might be co-expressed in patient-derived HNSCC cells, three cell lines were subjected to fluorescence-activated cell sorting (FACS) experiment based on their cell surface CD24+/CD44+ double positivity. CD24+/CD44+ double positivity was indicated recently to potentially mark for HNSCC cell population with stem cell like characteristics [45]. Also HNSCC patients with CD24 and CD44 double-positive cells showed the lowest overall survival rates [46]. Importantly, CIP2A has been shown to drive *in vivo* tumorigenesis and MYC expression in two of the studied cell lines, UT-SCC7 and UT-SCC9 [24], and all three cell lines do contain a population of cells that have characteristics of stem cell like cells as they are able to form spheres in serum free medium in low attachment plates (Sittig et al., unpublished results).

Cell sorting experiment revealed that all cell lines were 100% positive for CD44, which is in line with recent data that CD44 is expressed almost ubiquitously in HNSCC cells in culture [46]. Instead, CD24+/CD44+ double positivity did select for a side population of cells with frequency varying from 11% to 70% depending on the cell line (Fig. 4D, E, F). Indicative of increased stem cell like potential of CD44+/CD24+ double positive side population, these cells showed a clear enrichment in Oct4 expression as compared to CD44+ positive bulk of the cells (Fig. 4G, H, I). Importantly, although CIP2A expression pattern between two cell populations was more variable, Oct4 and CIP2A were co-expressed in the CD44+/CD24+ positive side population in all studied cell lines (Fig. 4G, H, I).

From these data we conclude that CIP2A and Oct4 are co-expressed in side-population of HNSCC cells and thus their regulatory relationship may contribute to HNSCC progression and therapy response.

### Oct4 positivity is linked to poor HNSCCC tumor differentiation level and increased radioresistance whereas CIP2A confers poor HNSCC patient survival

**Figure 5:**
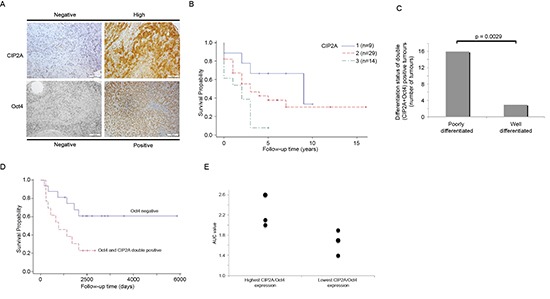
Double Oct4 and CIP2A positivity is linked to poor differentiation level and increased radioresistancy in HNSCC patients **(A)** Representative figures of negative and high CIP2A or Oct4 expression in HNSCC. White bar represents 100 μm. **(B)** The Kaplan–Meier 5 year overall survival curve from the HNSCC material (*n* = 52). The higher (blue) curve (1) represents the patients with a negative CIP2A expressing tumor, red curve (2) represents low CIP2A positivity and the lowest (green) curve (3) represents HNSCC patients having strong CIP2A expression. **(C)** The distribution as a percentage of poorly and well-differentiated tumours in Oct4/CIP2A double positive HNSCCs. **(D)** The Kaplan–Meier 5 year overall survival curve from the HNSCC patients treated with radiotherapy (*n* = 29). The higher (blue) curve represents the patients with Oct4 negative tumor and the lower (red) curve represents patients with double Oct4 and CIP2A positivity. **(E)** Relative Oct4/CIP2A mRNA expression values of six different HNSCC cell lines with highest and lowest double Oct4/CIP2A expression index were compared to radioresistancy expressed in area under the survival curve (AUC) values.

To clarify the possible clinical importance of CIP2A and Oct4 in HNSCC, we studied CIP2A and Oct4 expression in 52 head and neck squamous cell carcinoma (HNSCC) patient samples by immunohistochemistry (Fig. 5A). As shown in Table 2, 82.7% of HNSCC cancer samples were CIP2A positive, whereas 36.5% were Oct4 positive. Interestingly, Oct4 positive HNSCC tumors were always also CIP2A positive (Table 2). The relative expression levels of CIP2A and Oct4 were monitored, and their association to 5-year overall survival were studied in these 52 HNSCC patients. Staining intensity of CIP2A was categorized to three different groups (negative, low or high) whereas Oct4 exhibited either negative or positive staining in HNSCC tumours (Fig. 5A). Importantly, high CIP2A positivity indicated a reduced overall 5-year survival, compared to patients with low or negative CIP2A positivity (*P* = 0.020, log-rank test; Fig. 5B). Together with previously demonstrated essential tumor promoting function for CIP2A in HNSCCs [24], this data indicate that CIP2A is a driver oncoprotein in HNSCC. However, when overall survival of Oct4 positive cancers was compared to Oct4 negative cancers, a trend towards poor patient survival was observed in Oct4 positive cases, however this effect was not statistically significant (Supplementary Figure 4). Next, we set to study the other clinical variables that might be linked to cancers that are positive for both Oct4 and CIP2A, and assessed the differentiation status of the tumors, as low differentiation grade is known to be associated with existence of more stem-like cells [43]. Indeed, when histological grading of HNSCC samples was taken into account, we observed that 16/19 (84.2%) of Oct4/CIP2A double positive tumors were poorly differentiated whereas only 3/19 (15.8%) of CIP2A/Oct4 double positive were well-differentiated tumors (*p* = 0.0029; Fig. 5C). These results are in line with Oct4 function as a key regulator of stemness and cell differentiation.

**Table 2:** Immunopositivity of CIP2A and Oct4 expression in head and neck squamous cell carcinoma (HNSCC) patient samples

	CIP2A	Oct4	Oct4 and CIP2A Co-expression
*n*	52	52	52
Positive	43/52	19/52	19/52
%	82, 7	36, 5	36, 5

**Figure 6:**
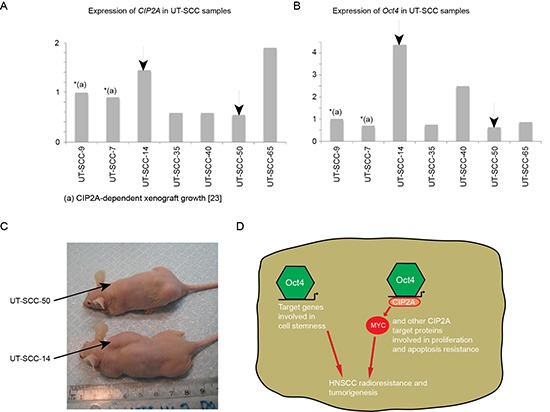
CIP2A and Oct4 double positivity is linked to cancer aggressiveness and increased radioresistance in HNSCC cell lines **(A-B)** Relative mRNA expression levels of CIP2A and Oct4 in six different HNSCC cell lines (UT-SCC) were studied with qPCR. Xenograft capacity demonstrates how easily HNSCC cell line is capable to grow in nude mice. ND = not determined. **(C)** Xenoraft experiment where HNSCC cell lines containing low (UT-SCC-50) and high (UT-SCC-14) Oct4/CIP2A mRNA expression levels were injected into nude mouse subcutaneous and followed for five weeks. **(D)** CIP2A expression is important for HNSCC tumorigenicity and radioresistancy. In the context of Oct4 positive cells with increased stemness, Oct4-driven CIP2A expression contributes to clinical radioresistance and tumorigenesis by affecting oncogenic pathways involved in proliferation and apoptosis resistance.

*In* vivo testicular irradiation experiment indicated that CIP2A and Oct4 were expressed in radioresistant population of spermatogonia (Fig. 1). Radiotherapy is widely used in HNSCC treatments, but the markers that would predict for radioresistancy in HNSCC are mostly lacking. Among the studied patient material, 29 of our 52 HNSCC patients (56%) were treated with radiotherapy. Among those, 55% of patients' tumors were negative for Oct4 expression, whereas 45% of patients' tumors were Oct4 positive and expressed also CIP2A. Importantly, in the radiotherapy group, Oct4 positivity predicted for a reduced overall 5-year survival, compared to patients with Oct4 negative staining (*P* = 0.027, log-rank test; Fig. 5D). To validate the clinical results, we compared Oct4 and CIP2A expression levels in UT-SCC cell lines evaluated in this study (Fig. 4B and 6A, B), to the intrinsic radioresistancy of these cell lines based on previous publications [47, 48]. When relative Oct4/CIP2A mRNA expression values of six different HNSCC cell lines with the highest and lowest double Oct4/CIP2A expression index were compared to the area under the survival curve values (AUC), a tendency between Oct4/CIP2A double positivity and the intrinsic radioresistancy was observed (Table 3, Fig. 5E).

**Table 3:** Relative CIP2A and Oct4 mRNA expression and logarithmic AUC radiosensitivity values of HNSCC cell lines

	Cell line	CIP2A/Oct4	AUC Mean log (+SD) [95% CI]
Highest	UT-SCC-24A	4.1	2.6 (0.3) [2.29–2.91]
	UT-SCC-15	4.9	2.1 (0.1) [2.00–2.21]
	UT-SCC-11	4.3	2.0 (0.2) [1.79–2.21]
Lowest	UT-SCC-19A	1.7	1.7 (0.1) [1.60–1.80]
	UT-SCC-9	1.4	1.4 (0.1) [1.30–1.50]
	UT-SCC-8	1.8	1.9 (0.1) [1.80–2.00]

Together these results demonstrate that CIP2A expression predicts for poor patient survival in HNSCC. Moreover, the results indicate that high Oct4 and CIP2A expression in HNSCC cells confer HNSCC tumour radioresistancy.

### Association of CIP2A and Oct4 status to *in vivo* aggressiveness of HNSCC cell lines

We previously demonstrated that xenograft growth of two of the HNSCC cell lines, UT-SCC-9 and UT-SCC-7 is very significantly inhibited by CIP2A depletion [24]. Moreover results above indicate that CIP2A expression correlates with tumor aggressiveness *in vivo.* To further test how CIP2A and Oct4 expression in HNSCC cells correlate with their tumorigenic capacity, we selected for subcutaneous xenograft experiment UT-SCC cell lines that contained either lower (UT-SCC-50) or higher (UT-SCC-14) CIP2A mRNA expression levels than in cell lines UT-SCC-9 and UT-SCC-7 whose CIP2A-dependence has been validated. Regarding Oct-4, UT-SCC14 had clearly higher expression than CIP2A-dependent cell lines, whereas expression in UT-SCC-50 was in a range of tumorigenic UT-SCC-7 cells (Fig. 6A and 6B). Notably, within five weeks, all mice (3/3) injected with UT-SCC-14 cells (CIP2A/Oct4 double positive) formed large palpable tumours (range 1.8-2.5 cm; Fig. 6C), whereas only 1/3 mice injected with low CIP2A expressing HNSCC cell line (UT-SCC-50) formed a barely detectable tumour (Fig. 6C).

These data further support our conclusions that CIP2A expression confers HNSCC tumorigenicity. We further postulate that in the context of Oct4 positive cells with increased stemness, Oct4-driven CIP2A expression contributes to clinical radioresistance in virtue by its effects on various oncogenic pathways rendering to increased proliferation and apoptosis resistance (Fig. 6D).

## DISCUSSION

Resistance towards DNA damaging anticancer therapies has been demonstrated to be associated with stem cell nature of cancer cells [49]. Oct4 is a stem cell transcription factor that is overexpressed in various human cancer types, and this expression has been linked to both poor patient survival and resistance to DNA-damaging therapies [22, 44]. In this work we identify CIP2A as a novel Oct4 target gene associated with cellular radioresistance and tumorigenicity. The radioresistance phenotype of Oct4/CIP2A double positive cells is indicated by various lines of evidence extending from radioresistant normal testicular cell population *in vivo* (Fig. 1C), to both HNSCC tumor tissues (Fig. 5D) and cell lines (Fig. 5E).

Capacity of either normal or malignant tissue to recover from DNA damaging insult is dependent on degree of DNA damage induced, cellular capacity to repair the damaged DNA, as well as capacity of cells to proliferate during the regeneration phase. In the context of malignant tumors, this equals to regrowth of the tumor following therapy. Traditionally, the role of Oct4 and other *bona fide* stem cell factors in radioresistance has been linked to their function in maintaining DNA damage resistant pool of stem cells, that then give rise to regenerating pool of progenitor cells. However, Oct4 target genes that contribute to cell proliferation and apoptosis resistance are poorly understood. In that regard, identification of CIP2A as a novel Oct4 target gene may have important implication in our understanding the mechanisms by which high Oct4 expression drives cellular radioresistance beyond its role in regulating cell stemness (Fig. 6D). In addition to MYC, CIP2A mediated regulation of PP2A serine/threonine phosphatase activity promotes activity of several oncogenic mechanisms such as Akt kinase activity and E2F1 phosphorylation [27, 28]. Therefore it will be of great interest to further study whether activities of these CIP2A effector pathways are regulated by Oct4, and what is their functional relevance for Oct4 driven radioresistance. In summary our data extends the function of Oct4 from being only a regulator of cell stemness, to regulator of phosphoprotein signaling via CIP2A, and we postulate that combination of these activities contribute to clinical radioresistance (Fig. 6D).

CIP2A promotes malignant cell transformation and tumour growth [24, 25], and importantly its downregulation does not cause detrimental systemic side effects *in vivo* [29]. As CIP2A is overexpressed practically in all human cancer types, and its expression predicts poor patient survival in a dozen different cancers [25], it is an obvious drug target candidate protein. It is also evident that future identification of target mechanisms regulated by Oct4 driven CIP2A expression may help in development of novel radiosensitation therapies. In that regard, results of this study may have clinical potential in treatment of many different malignancies, in addition to HNSCC.

Head and neck cancers are the sixth commonest cancer types worldwide with poor prognosis [1]. In order to study the importance of Oct4 and CIP2A co-expression in other than testicular cancers, we used HNSCC cell lines and patient specimens. Our results demonstrate that CIP2A and Oct4 expression is linked in HNSCC cell lines, and that the cell lines with CIP2A and Oct4 co-expression were radioresistant. In patient samples of HNSCC, CIP2A was expressed in 82.7% and Oct4 in 36.5% of the studied tumor samples, respectively, and all Oct4 positive HNSCC tumours were also CIP2A positive. Our demonstration that CIP2A and Oct4 expression is linked to poor differentiation level of HNSCC tumors, and predicts for a poor patient survival among HNSCC patients treated with radiotherapy is intriguing. It is clear further prospective study using larger HNSCC patient groups is warranted to validate the potential clinical usefulness of these results. In general terms, our results indicate that cancer cells can adapt similar mechanisms for X-irradiation resistance as normal stem cells, and that this might be one explanation for poor radiotherapy response in those cancer cases. Consequently, targeting of the mechanisms implicated in radioresistance could provide a foundation for new radiosensitation therapies. CIP2A inhibition has previously been shown to potently inhibit HNSCC tumorigenesis [24], whereas here we demonstrate that high CIP2A expression was linked to poor overall 5-year survival in HNSCC. Together these results support the idea that targeting of CIP2A could simultaneously be used for radiosensitation of Oct4 positive cancer stem cell-like population, as well as for eradication of the Oct4 negative bulk of the HNSCC tumor.

## MATERIALS AND METHODS

### *In vivo* X-irradiation and tumor formation experiments

Mice were housed in plastic cages (Tecniplast, Buguggiate, Italy) in a climate-controlled room at the Animal Centre of Turku University (Turku, Finland). Aspen chips (Tapvei Co., Kaavi, Finland) were used as bedding material. Animals were maintained on a 12 h light/12 h dark cycle (lighted from 07 to 19 h) and they had free access to tap water and standard laboratory animal feed (Commercial RM3 (E) SQC, Special Diet Service, Witham, UK). Two-month-old mice (C57BL/6) were anaesthetised with 2.5% Avertin (Aldrich Chemical Co., Milwaukee, WI, USA) i.p. and locally irradiated (with a water-equivalent build-up layer, focus-target distance 100 cm, field size 4 × 10 cm, dose rate 3 Gy/min) by 3-4 Gy using 6 MV X-rays produced by a Clinac 600C linear accelerator (Varian, Palo Alto, CA, USA). The mice were sacrificed by neck dislocation under CO_2_ anaesthesia 6, 17, 24, 48, 72, 96 or 144 hours after X-irradiation and their testes were dissected and decapsulated. Seminiferous tubules were snap-frozen in liquid nitrogen and used in RNA analyses. Radiation dose in testis was determined mathematically using a computer tomography based Eclipse planning system (Varian, Palo Alto, CA, USA). Control mice were subjected to the same treatment omitting the X-irradiation. In xenograft experiment previously established UT-SCC-14 (originating from a persistent T3N1M0 Gr 2 cancer of the mobile tongue) and UT-SCC-50 (established from a recurrent T2N0M0 Gr 3 glottic laryngeal tumor) HNSCC cell lines [50] were seleceted for *in vivo* tumour formation experiment. 2 × 10^6^ cells were injected into immunocompromised mouse subcutaneously. Altogether 6 mice were injected and the size of the palpable tumors was evaluated every third day for five weeks. All animal experiments were conducted in accordance with the guidelines of the Provincial Government of Southern Finland and handled in accordance with the institutional animal care policies of the University of Turku. The Experimental Animal Committee of the University of Turku has approved all protocols used in animal experiments (ESLH-2007-08517).

### RNA isolation and cDNA synthesis

Total RNA was isolated from cell pellets or seminiferous tubules of mouse testis using Trisure reagent (Bioline, London, UK) according to the manufacturer's instructions. After isolation, RNA concentration was measured using a NanoDrop device (ND-1000; NanoDrop Technologies, Wilmington, DE, USA) and the RNA sample was run on agarose gel to confirm good quality of the isolated RNA (intact 28S and 18S ribosomal RNA bands). One microgram of RNA was processed further. Firstly, traces of contaminating genomic DNA were removed by treating the samples with DNase I (Invitrogen, Carlsbad, CA, USA). DyNAmo SYBR Green 2-step qRT-PCR Kit (Finnzymes, Espoo, Finland) was used for cDNA synthesis and 0.5 μg of template RNA was reverse-transcribed in a 20-μl-reaction with oligo(dT) primers while another 0.5 μg was used as a template in RT-reaction.

### Real-time PCR

Primers (Supplementary table 1) were designed to be located to different exonic sequences with the help of online Primer 3 software (http://frodo.wi.mit.edu/) and mRNA sequence data available at Ensembl (www.ensembl.org/) and NCBI (http://www.ncbi.nlm.nih.gov/) databases to avoid amplification of genomic DNA. To avoid misleading detection of Oct4, the primers were designed so that they do not recognize any of the Oct4 pseudogenes [41]. Amplification of target cDNAs was performed using CFX96 real-time PCR detection system device (Bio-Rad Laboratories Inc., Hercules, CA, USA) and the DyNAmo Flash SYBR green qPCR kit (F-415L; Finnzymes, Espoo, Finland) according to the manufacturers' instructions. Quantitative real-time PCR was executed under the following conditions: 95°C for 7 min followed by 40 cycles of 94°C for 1 s and 55–64°C (depending on the primer pair; see Supplementary table 1) for 15s. Relative gene expression data was normalized to expression level of endogenous house-keeping genes (*Ppia* (cyclophilin A) and *RPL19* (ribosomal protein L19) using 2^-ΔΔC(t) method [51]. Specificity of PCR reactions was verified by agarose gel electrophoresis and melting curve analysis. One band of the expected size and a single peak, respectively, were required.

### Antibodies, immunohistochemistry and tissue samples

Following antibodies were used for Western blotting: CIP2A (CIP2A (2610-3B5) sc-80659, mouse monoclonal, 1:1000, Santa Cruz Biotechnology), Oct4 (Oct-3/4 (c-10), sc-5279, mouse monoclonal, 1:1000, Santa Cruz Biotechnology), p-myc (anti-cMyc phospho-Ser62, monoclonal mouse (33A12E10), 1:1000) and actin (anti-β-actin Clone AC-74, monoclonal mouse, Sigma-Aldrich 1:5000). Formalin-fixed, paraffin-embedded sections of mouse and human organs were cut into 6 μm thin sections, deparaffinised and thereafter rehydrated. Epitope retrieval was then proceeded in 10 mM Tris-EDTA-buffer (pH 9) during 4 min in microwave oven 4 min 850 W followed by 15 min at a lower power (150 W). After blocking with 3% BSA PBS for 10 min the slides were rinsed in Tris-HCl (pH 7.4), and incubated overnight with primary antibodies against CIP2A (1:10000 rabbit polyclonal anti-CIP2A [52], Oct4 (1:200 mouse monoclonal, sc-5279 Santa Cruz), ki-67 (1:5000 mouse monoclonal anti-ki67 (M7240, Dako)), or c-Myc (1:200 mouse monoclonal 9E10 (Nordic Biosite). Control slides were incubated with normal nonimmunized appropriate animal serum. The samples were then incubated appropriate secondary antibody (Dako EnVision anti-rabbit or anti-mouse) for 30 min and 10 min in DAB+ liquid Dako (K3468). The usage of human tissue samples was approved by the Finnish national authority for medicolegal affairs (Dnro 889/04/047/08) and regional ethics committee of University of Turku (Dnro 146/2007).

### Cell sorting

UT-SCC2, -7 and -9 cells were harvested with 0.01% Trypsin-EDTA and washed twice with cold buffer (D-PBS, 2% FCS, 0.01% sodium azide). Primary antibodies (anti-human CD44 (clone 9B5) rat monoclonal antibody was a kind gift from Professor Marko Salmi (Turku, Finland), anti-human CD24 (clone ML5) Alexa Fluor® 647 mouse monoclonal antibody (BD Biosciences)) were added at dilution of 1:100 and incubated for 1 hour at +4°C, after which cells were washed. Secondary antibody (Alexa Fluor® 488 Goat Anti-Rat IgG (Life Technologies)) was added at dilution of 1:400 and incubated for 1 hour at +4°C. The cells were washed and sorted with BD FACSAria™ III cell sorter (BD Biosciences). After sorting the cells were lysed with TXLB buffer. Sorting experiment was repeated three times for each cell line.

### siRNA transfections

Tcam2 and Tera1 cell lines were cultured in RPMI with Glutamax (Invitrogen, 61870-010), 10% FCS and antibiotics (streptomycin and penicillin) and were adapted to 50–250 nM concentration of CIP2A or scramble (SCR) siRNA or medium (negative control). siRNA was transfected with Oligofectamine reagent (Invitrogen) according to the manufacturer's instructions. siRNA were used in 50–250 nM concentration for 3 d or 5 d. Used siRNA sequences are presented in Supplementary table 2.

### Promoter assay

Tcam2-cells were double transfected using the Surefect transfection reagent according to manufactures protocol (Nunclon Surface, Nunc). Cells were transfected with CIP2A-promoter construct (1802 bp upstream [38], renilla plasmid and siRNA (either scrambled or siOct4-1). Promoter construct (200 ng), renilla (10 ng) and 2 pmol of siRNA were transfected per 96 well plate. Transfections with −1802 bp, −865 bp and −1802ΔCIP2ALuc CIP2A promoter constructs were also done as described above only without siRNAs. −1802ΔCIP2ALuc construct was produced by GenScript mutagenesis service from −1802CIP2ALuc promoter construct and resulting promoter sequence was validated by DNA sequencing. After 3 days the promoter activity was measured using Promega's Dual-Glo luciferase Assay system (E2940) according to manufactures protocol. Luminescence was measured with Victor-multilabel counter 1420 (PerkinElmer).

### Murine embryonic stem cell (mESC) *in vitro* studies

ZHBTc4 ES cells [38] were kindly provided by Dr. Hitoshi Niwa (Center For Developmental Biology, Laboratory for Pluripotent Cell Studies, Kobe, Japan). Murine ESCs were kept in undifferentiated state by culturing them on a feeder layer of mitomycin C-inactivated mouse embryonic fibroblasts with basic ES cell medium. The cells were passaged every two-three days and ES cell medium was exchanged daily. To study the effect of Oct4-mediated differentiation of mES cells ZHBTc4 ES cells were plated on to 0.1% gelatin-coated culture dishes and treated without or with 1 μg/ml doxycycline. Samples were collected 6, 12, 24, 48 and 72 hours by scraping off the cells, pelleting them by centrifugation and snap-freezing them in liquid nitrogen. Three independent experiments were performed, all of which gave similar results.

### Derivation of embryonic stem cells from blastocysts

Embryonic stem cells (ESCs) were isolated from CIP2A and WT mouse blastocysts as described by Bryja and coworkers [53]. Briefly, time-mated females were killed at E3.5, and the blastocysts were flushed out of the uterine horn. Blastocysts were plated to dishes containing mitotically inactivated feeder cells (mouse embryonic fibroblasts, MEFs). Blastocysts were allowed to attach to MEFs and grow in ES medium containing knockout serum replacement (SR-ES medium). The content of the medium was: Knockout DMEM supplemented with 20% Knockout SR (Gibco), penicillin (100 U/ml)/streptomycin (100 g/ml) (Gibco), 2 mM L-glutamine (Gibco), 1 X minimal essential medium nonessential amino acids (Gibco), 100 μM-mercaptoethanol and recombinant mouse leukemia inhibitory factor (1,000 U/ml of ESGRO, Chemicon International, Temecula, CA). The blastocysts and ESCs derived from the inner cell mass of blastocysts were allowed to grow alternately in SR-ES medium and FCS-ES medium. In FCS-ES medium SR was replaced by 20% fetal calf serum (FCS). In the method, the cells were grown always after trypsinization in FCS-ES for one day to allow greater trophic support, whereas SR-ES medium supported selective propagation of ESCs between trypsinizations.

### Statistical methods

The results were analysed for statistically significant differences using one-way analysis of variance, followed by Dunnett's tests (vs. 0h) for multiple comparisons of independent groups of samples (Fig. 1C). Student's t-test was used to compare mRNA levels of *CIP2A, Nanog* and *Oct4* in blastocyst-derived ES cells between WT and CIP2A HOZ mice (Supplementary Fig. 2). The assumptions of normal distribution and equal variance within the data sets were fulfilled. Correlation of gene expression levels in UT-SCC cell lines was analysed by using linear regression analysis (Fig. 4B–C). In HNSCC patient data statistical analyses were presented using frequencies and percents. The differences between CIP2A and Oct4 expression to 5-year overall survival were studied using survival analysis. Survival curves were estimated using Kaplan-Meier technique, and differences were tested using a log-rank test. *p*-values (two-tailed) less than 0.05 were considered statistically significant (Fig. 5B and 5D). For each UT-SCC cell line 95% confidence intervals were calculated for AUC means (Table 3, Fig. 5E). Statistical analyses were performed using the SAS System for Windows, Version 9.3 (SAS Institute Inc, Cary, NC, USA).

## SUPPLEMENTARY FIGURES AND TABLES



## References

[1] Ferlay J, Shin HR, Bray F, Forman D, Mathers C, Parkin DM (2010). Estimates of worldwide burden of cancer in 2008: GLOBOCAN 2008. Int J Cancer.

[2] Ma J, Liu Y, Huang XL, Zhang ZY, Myers JN, Neskey DM, Zhong LP (2012). Induction chemotherapy decreases the rate of distant metastasis in patients with head and neck squamous cell carcinoma but does not improve survival or locoregional control: a meta-analysis. Oral oncology.

[3] Agrawal N, Frederick MJ, Pickering CR, Bettegowda C, Chang K, Li RJ, Fakhry C, Xie TX, Zhang J, Wang J, Zhang N, El-Naggar AK, Jasser SA, Weinstein JN, Treviño L, Drummond JA (2011). Exome sequencing of head and neck squamous cell carcinoma reveals inactivating mutations in NOTCH1. Science.

[4] Lui VW, Hedberg ML, Li H, Vangara BS, Pendleton K, Zeng Y, Lu Y, Zhang Q, Du Y, Gilbert BR, Freilino M, Sauerwein S, Peyser ND, Xiao D, Diergaarde B, Wang L (2013). Frequent mutation of the PI3K pathway in head and neck cancer defines predictive biomarkers. Cancer discovery.

[5] Pickering CR, Zhang J, Yoo SY, Bengtsson L, Moorthy S, Neskey DM, Zhao M, Ortega Alves MV, Chang K, Drummond J, Cortez E, Xie TX, Zhang D, Chung W, Issa JP, Zweidler-McKay PA (2013). Integrative genomic characterization of oral squamous cell carcinoma identifies frequent somatic drivers. Cancer discovery.

[6] Kokko LL, Hurme S, Maula SM, Alanen K, Grenman R, Kinnunen I, Ventela S (2011). Significance of site-specific prognosis of cancer stem cell marker CD44 in head and neck squamous-cell carcinoma. Oral oncology.

[7] Sano D, Matsumoto F, Valdecanas DR, Zhao M, Molkentine DP, Takahashi Y, Hanna EY, Papadimitrakopoulou V, Heymach J, Milas L, Myers JN (2011). Vandetanib restores head and neck squamous cell carcinoma cells' sensitivity to cisplatin and radiation *in vivo* and *in vitro*. Clin Cancer Res.

[8] Moeller BJ, Yordy JS, Williams MD, Giri U, Raju U, Molkentine DP, Byers LA, Heymach JV, Story MD, Lee JJ, Sturgis EM, Weber RS, Garden AS, Ang KK, Schwartz DL (2011). DNA repair biomarker profiling of head and neck cancer: Ku80 expression predicts locoregional failure and death following radiotherapy. Clin Cancer Res.

[9] Visvader JE, Lindeman GJ (2008). Cancer stem cells in solid tumours: accumulating evidence and unresolved questions. Nat Rev Cancer.

[10] Chen YS, Huang WL, Chang SH, Chang KW, Kao SY, Lo JF, Su PF (2013). Enhanced filopodium formation and stem-like phenotypes in a novel metastatic head and neck cancer cell model. Oncology reports.

[11] Burdon T, Smith A, Savatier P (2002). Signalling, cell cycle and pluripotency in embryonic stem cells. Trends in cell biology.

[12] Cheng L, Sung MT, Cossu-Rocca P, Jones TD, MacLennan GT, De Jong J, Lopez-Beltran A, Montironi R, Looijenga LH (2007). OCT4: biological functions and clinical applications as a marker of germ cell neoplasia. J Pathol.

[13] Nichols J, Zevnik B, Anastassiadis K, Niwa H, Klewe-Nebenius D, Chambers I, Schöler H, Smith A (1998). Formation of pluripotent stem cells in the mammalian embryo depends on the POU transcription factor Oct4. Cell.

[14] Boiani M, Schöler HR (2005). Regulatory networks in embryo-derived pluripotent stem cells. Nat Rev Mol Cell Biol.

[15] Sinha N, Mukhopadhyay S, Das DN, Panda PK, Bhutia SK (2013). Relevance of cancer initiating/stem cells in carcinogenesis and therapy resistance in oral cancer. Oral oncology.

[16] Monk M, Holding C (2001). Human embryonic genes re-expressed in cancer cells. Oncogene.

[17] Ezeh UI, Turek PJ, Reijo RA, Clark AT (2005). Human embryonic stem cell genes OCT4, NANOG, STELLAR, and GDF3 are expressed in both seminoma and breast carcinoma. Cancer.

[18] Chiou SH, Wang ML, Chou YT, Chen CJ, Hong CF, Hsieh WJ, Chang HT, Chen YS, Lin TW, Hsu HS, Wu CW (2010). Coexpression of Oct4 and Nanog enhances malignancy in lung adenocarcinoma by inducing cancer stem cell-like properties and epithelial-mesenchymal transdifferentiation. Cancer Res.

[19] Ponti D, Costa A, Zaffaroni N, Pratesi G, Petrangolini G, Coradini D, Pilotti S, Pierotti MA, Daidone MG (2005). Isolation and *in vitro* propagation of tumorigenic breast cancer cells with stem/progenitor cell properties. Cancer Res.

[20] Marie-Egyptienne DT, Lohse I, Hill RP (2013). Cancer stem cells, the epithelial to mesenchymal transition (EMT) and radioresistance: potential role of hypoxia. Cancer Lett.

[21] Chiou SH, Yu CC, Huang CY, Lin SC, Liu CJ, Tsai TH, Chou SH, Chien CS, Ku HH, Lo JF (2008). Positive correlations of Oct-4 and Nanog in oral cancer stem-like cells and high-grade oral squamous cell carcinoma. Clin Cancer Res.

[22] Shen L, Huang X, Xie X, Su J, Yuan J, Chen X (2014). High Expression of SOX2 and OCT4 Indicates Radiation Resistance and an Independent Negative Prognosis in Cervical Squamous Cell Carcinoma. The journal of histochemistry and cytochemistry : official journal of the Histochemistry Society.

[23] Zhang M, Kumar B, Piao L, Xie X, Schmitt A, Arradaza N, Cippola M, Old M, Agrawal A, Ozer E, Schuller DE, Teknos TN, Pan Q (2014). Elevated intrinsic cancer stem cell population in human papillomavirus-associated head and neck squamous cell carcinoma. Cancer.

[24] Junttila MR, Puustinen P, Niemela M, Ahola R, Arnold H, Bottzauw T, Ala-aho R, Nielsen C, Ivaska J, Taya Y, Lu SL, Lin S, Chan EK, Wang XJ, Grenman R, Kast J (2007). CIP2A inhibits PP2A in human malignancies. Cell.

[25] Khanna A, Pimanda JE, Westermarck J (2013). Cancerous inhibitor of protein phosphatase 2A, an emerging human oncoprotein and a potential cancer therapy target. Cancer Res.

[26] De P, Carlson J, Leyland-Jones B, Dey N (2014). Oncogenic nexus of cancerous inhibitor of protein phosphatase 2A (CIP2A): an oncoprotein with many hands. Oncotarget.

[27] Khanna A, Pimanda JE, Westermarck J (2013). Cancerous Inhibitor of Protein Phosphatase 2A (CIP2A), an emerging human oncoprotein and a potential cancer therapy target Cancer Research.

[28] Laine A, Sihto H, Come C, Rosenfeldt MT, Zwolinska A, Niemela M, Khanna A, Chan EK, Kahari VM, Kellokumpu-Lehtinen PL, Sansom OJ, Evan GI, Junttila MR, Ryan KM, Marine JC, Joensuu H (2013). Senescence Sensitivity of Breast Cancer Cells Is Defined by Positive Feedback Loop between CIP2A and E2F1. Cancer discovery.

[29] Ventelä S, Côme C, Mäkelä JA, Hobbs RM, Mannermaa L, Kallajoki M, Chan EK, Pandolfi PP, Toppari J, Westermarck J (2012). CIP2A promotes proliferation of spermatogonial progenitor cells and spermatogenesis in mice. PLoS ONE.

[30] Kanatsu-Shinohara M, Inoue K, Lee J, Yoshimoto M, Ogonuki N, Miki H, Baba S, Kato T, Kazuki Y, Toyokuni S, Toyoshima M, Niwa O, Oshimura M, Heike T, Nakahata T, Ishino F (2004). Generation of pluripotent stem cells from neonatal mouse testis. Cell.

[31] Ko K, Tapia N, Wu G, Kim JB, Bravo MJ, Sasse P, Glaser T, Ruau D, Han DW, Greber B, Hausdörfer K, Sebastiano V, Stehling M, Fleischmann BK, Brüstle O, Zenke M (2009). Induction of pluripotency in adult unipotent germline stem cells. Cell stem cell.

[32] Ventela S, Makela JA, Kulmala J, Westermarck J, Toppari J (2012). Identification and regulation of a stage-specific stem cell niche enriched by Nanog-positive spermatogonial stem cells in the mouse testis. Stem cells (Dayton, Ohio).

[33] Zhou Q, Li Y, Nie R, Friel P, Mitchell D, Evanoff RM, Pouchnik D, Banasik B, McCarrey JR, Small C, Griswold MD (2008). Expression of stimulated by retinoic acid gene 8 (Stra8) and maturation of murine gonocytes and spermatogonia induced by retinoic acid *in vitro*. Biology of reproduction.

[34] Giuili G, Tomljenovic A, Labrecque N, Oulad-Abdelghani M, Rassoulzadegan M, Cuzin F (2002). Murine spermatogonial stem cells: targeted transgene expression and purification in an active state. EMBO reports.

[35] Dym M, Jia MC, Dirami G, Price JM, Rabin SJ, Mocchetti I, Ravindranath N (1995). Expression of c-kit receptor and its autophosphorylation in immature rat type A spermatogonia. Biology of reproduction.

[36] Clark AT (2007). The stem cell identity of testicular cancer. Stem cell reviews.

[37] Khanna A, Okkeri J, Bilgen T, Tiirikka T, Vihinen M, Visakorpi T, Westermarck J (2011). ETS1 mediates MEK1/2-dependent overexpression of cancerous inhibitor of protein phosphatase 2A (CIP2A) in human cancer cells. PLoS ONE.

[38] Niwa H, Miyazaki J, Smith AG (2000). Quantitative expression of Oct-3/4 defines differentiation, dedifferentiation or self-renewal of ES cells. Nat Genet.

[39] Junttila MR, Westermarck J (2008). Mechanisms of MYC stabilization in human malignancies. Cell Cycle.

[40] Niemela M, Kauko O, Sihto H, Mpindi JP, Nicorici D, Pernila P, Kallioniemi OP, Joensuu H, Hautaniemi S, Westermarck J (2012). CIP2A signature reveals the MYC dependency of CIP2A-regulated phenotypes and its clinical association with breast cancer subtypes. Oncogene.

[41] Liedtke S, Stephan M, Kögler G (2008). Oct4 expression revisited: potential pitfalls for data misinterpretation in stem cell research. Biol Chem.

[42] Bockelman C, Hagstrom J, Makinen LK, Keski-Santti H, Hayry V, Lundin J, Atula T, Ristimaki A, Haglund C (2011). High CIP2A immunoreactivity is an independent prognostic indicator in early-stage tongue cancer. British journal of cancer.

[43] Albers AE, Chen C, Köberle B, Qian X, Klussmann JP, Wollenberg B, Kaufmann AM (2012). Stem cells in squamous head and neck cancer. Crit Rev Oncol Hematol.

[44] Tsai LL, Yu CC, Chang YC, Yu CH, Chou MY (2011). Markedly increased Oct4 and Nanog expression correlates with cisplatin resistance in oral squamous cell carcinoma. J Oral Pathol Med.

[45] Han J, Fujisawa T, Husain SR, Puri RK (2014). Identification and characterization of cancer stem cells in human head and neck squamous cell carcinoma. BMC cancer.

[46] Oliveira LR, Oliveira-Costa JP, Araujo IM, Soave DF, Zanetti JS, Soares FA, Zucoloto S, Ribeiro-Silva A (2011). Cancer stem cell immunophenotypes in oral squamous cell carcinoma. J Oral Pathol Med.

[47] Pekkola-Heino K, Servomaa K, Kiuru A, Grenman R (1998). Sublethal damage repair capacity in carcinoma cell lines with p53 mutations. Head Neck.

[48] Farnebo L, Jerhammar F, Ceder R, Grafström RC, Vainikka L, Thunell L, Grénman R, Johansson AC, Roberg K (2011). Combining factors on protein and gene level to predict radioresponse in head and neck cancer cell lines. J Oral Pathol Med.

[49] Bao S, Wu Q, McLendon RE, Hao Y, Shi Q, Hjelmeland AB, Dewhirst MW, Bigner DD, Rich JN (2006). Glioma stem cells promote radioresistance by preferential activation of the DNA damage response. Nature.

[50] Pekkola-Heino K, Kulmala J, Klemi P, Lakkala T, Aitasalo K, Joensuu H, Grenman R (1991). Effects of radiation fractionation on four squamous cell carcinoma lines with dissimilar inherent radiation sensitivity. J Cancer Res Clin Oncol.

[51] Livak KJ, Schmittgen TD (2001). Analysis of relative gene expression data using real-time quantitative PCR and the 2(−Delta Delta C(T)) Method. Methods.

[52] Soo Hoo L, Zhang JY, Chan EK (2002). Cloning and characterization of a novel 90 kDa ‘companion’ auto-antigen of p62 overexpressed in cancer. Oncogene.

[53] Bryja V, Bonilla S, Cajánek L, Parish CL, Schwartz CM, Luo Y, Rao MS, Arenas E (2006). An efficient method for the derivation of mouse embryonic stem cells. Stem cells (Dayton, Ohio).

