# Supplementary material for: CIP2A is an Oct4 target gene involved in head and neck squamous cell cancer oncogenicity and radioresistance
Source: Oncotarget. 2014 Dec 5;6(1):144–58. doi: 10.18632/oncotarget.2670 (PMC4381584; doi:10.18632/oncotarget.2670)
Supplement: Supplementary file 1 [file oncotarget-06-144-s001.pdf]

SUPPLEMENTARY FIGURES AND TABLES

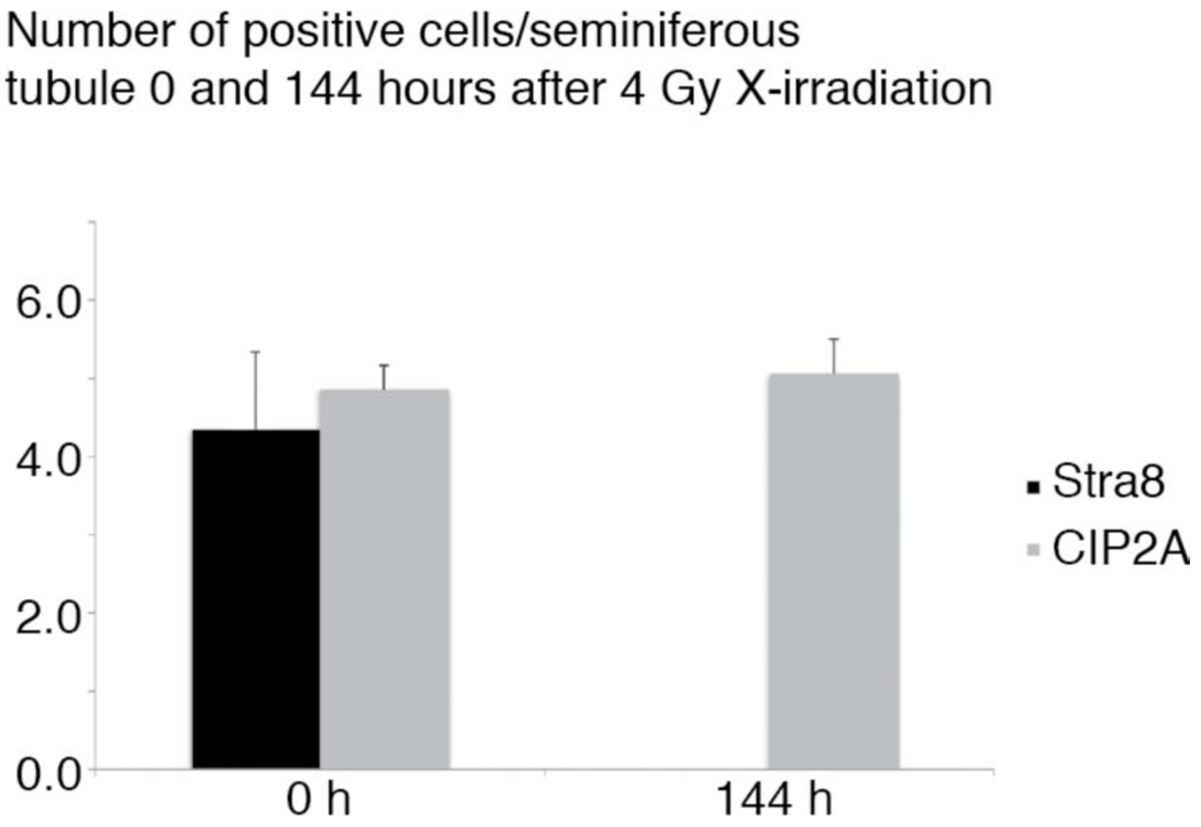

Supplementary Figure S1: Number of Stra8 and CIP2A positive cells/seminiferous tubule 0 and 144 hours after 4 Gy X-irradiation.

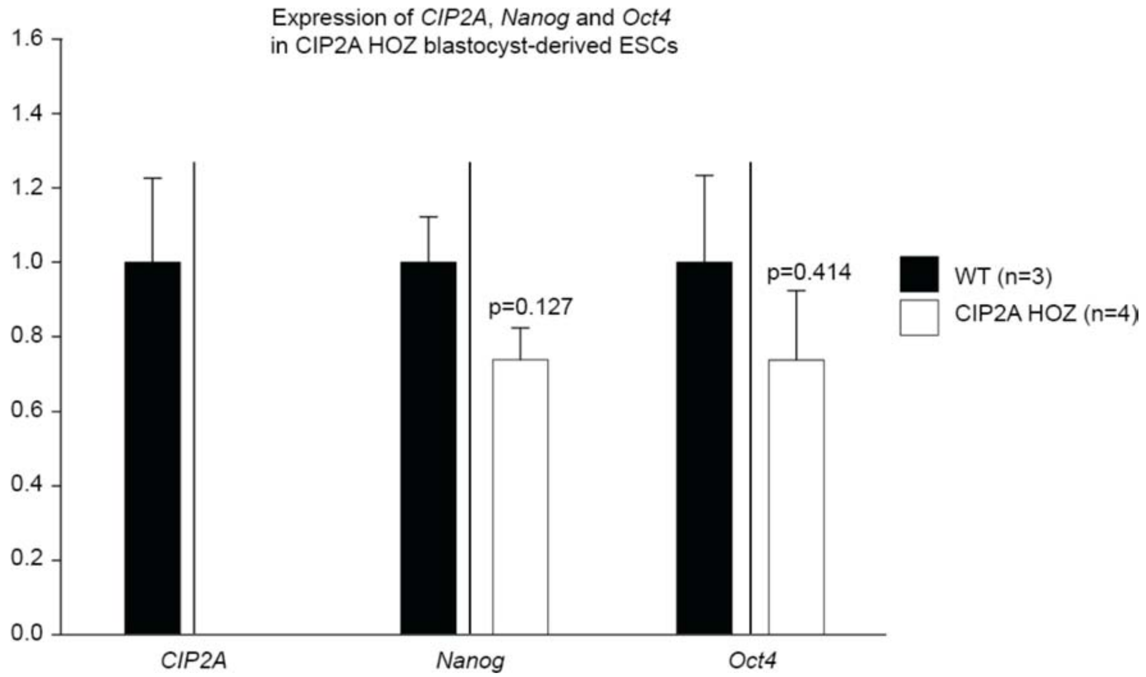

Supplementary Figure S2: mRNA expression of CIP2A, Nanog and Oct4 in CIP2A HOZ and wild type blastocyst-derived embryonic stem cells.

```
Database:  TRANSFAC MATRIX TABLE, Rel.3.3 06-01-1998
 Query:     CIP2A promoter
 Taxonomy:  Vertebrate
 Threshold: 90.0 poi


ATGTTGCTTT CTTAATGAGG TCACCTTCCC ATTTCCCTTA
TCAATTACCA entry        score
             -------------->
<A
HREF=http://www.cbrc.jp/htbin/bget_tfmatrix?M00137>M0
0137</A> Oct-1   93.1
                                          &lt;--------
------    <A
```

```
-1650
ATGTTGCTTTCTTAATGAGGTCACCTTCCCATTTCCCTTATCAATTACCAACATTATT
CTAGAGTAATAACGTCTATTTTTCTCTTTATATCATTTTCCTTACTTATTTATTGTG
TTTATTATTTGGTTCCCTACCAAAATGTAAGTTGCATGCCAGTAGTATTTTTGTTTT
TTTCACTGAGTCTATTGTGTCTAGAAAAGTGCTTAGCACATTGTAGATTCTCAATGA
ACTACTTATTGAATGAACAGTCCTATGAACCAGGTATCTCCCTTGGCCAGATTTTACC
TAATGAAGATTCTGCAGCAGTGAGAACTTGCCTAGAGTCACATCGTCAAAGGTGGAG
CTAGAATCTGTAAGCAACCTGGCTCTCTACTCTTTACCACTGCTGCATGTTACTGCAT
GGTGCCTCTCATTTATGTGGTGAATTTCAAAAGTACTATTTTGTATGTTTCCCTTAC
TAGACAGTTCCCTCGCAGCAGGGGATACTACTTCATCTCTGTTCCCTGACTGGCAGTT
TCTAACCCATGTTACACGCTCTATAATTGTTTACGGAATGAATGAAAGCATAAATGA
ACACACAAGCTACCCAAAGATTTTTACATGAATGTCCCAACTGGTTAAATCATTTTT
ATCTTTCAGATTTAAACTTTTATAAAAAACAAAACCAAAACACCAAACCTGTCTTCA
AAAATCTAGTAATTCAAAAAAATTCTTTTGAAGTGAAAAAACATGATTTCTGATAA
ACGATTTAATTAAATGGAAATGTACCCTATTCTTACAATTTCTACATCCTGGTTTTT
AAAGCTTTATTCGACGTTAGGAAATATTAAGCAAAAAGCTCCAAAACCAGTAACTGA
CTCGCTTCATTACATACACATGCAATAATATTAAGCTATCTCCGATTTAAAAAACGC
AAATAATAGTGCCATTTTCGTAGACATCGAGAATTATAAAACAATCAATCATGCCAG
AGAGAAACAGACACCTTCACTTACAAAAACTGACGCTAACGAGTAACACCCTCCGGA
GCAATACCTGGCCCAGGGTCAGCAGGAGGTAGTCTTCTCGAATAAATGTGACAAATA
CTTTGGTCAGGGCATGACTAATTTGCCACATAATGCCAATAGGTTGACTCTGAGAAA
CTACCATCCCCGGGCAGAAGGGGCGGGTGTAGGAGAGATTTAAAAACGAGGCCCCTC
CCAGGCCGCCTCAAAATCTAGGGCCCAAGATTCTTCTCCTCAGAGCTCCTCACCGTGT
CTAGACTAGGGGCAAGCGACCATTTCTCAGGTAGAGGATGACGCACAAACGAAAAAC
TGGCCATCCAACATGAAGGACGAGGAAGCGCACGAAATCAGAGCGCACAGCTGAGTG
AGGAATCCCCCTTCTCCTAACCGATTCCTCTCCCGAGAAATCGCGAGATTTCTCGCCT
TCACGGGATCTCAGGCCGAAACCTCGCGGCCTCTCAGACGAGGGTGGGTTAGCGGGG
GCAGCTCCCAACCCCCGTCCTGGACCCACAAATCACCTCGACCCCTGGCCCACCCCGGC
CGTCACCGAGAACGGTCCCCTAGGGTGCCTAGGGACTTCCGGAGCCCGACCGGATCCG
GAAGCTTCTGAGAGCGAGGGGGTGGGGCCGAAAATCAAAAAAAGC+1
```

Supplementary Figure S3: Bioinformatics analysis of -1802 bp CIP2A promoter fragment identified putative octamer binding elements at region -1650 to -1600 bp.

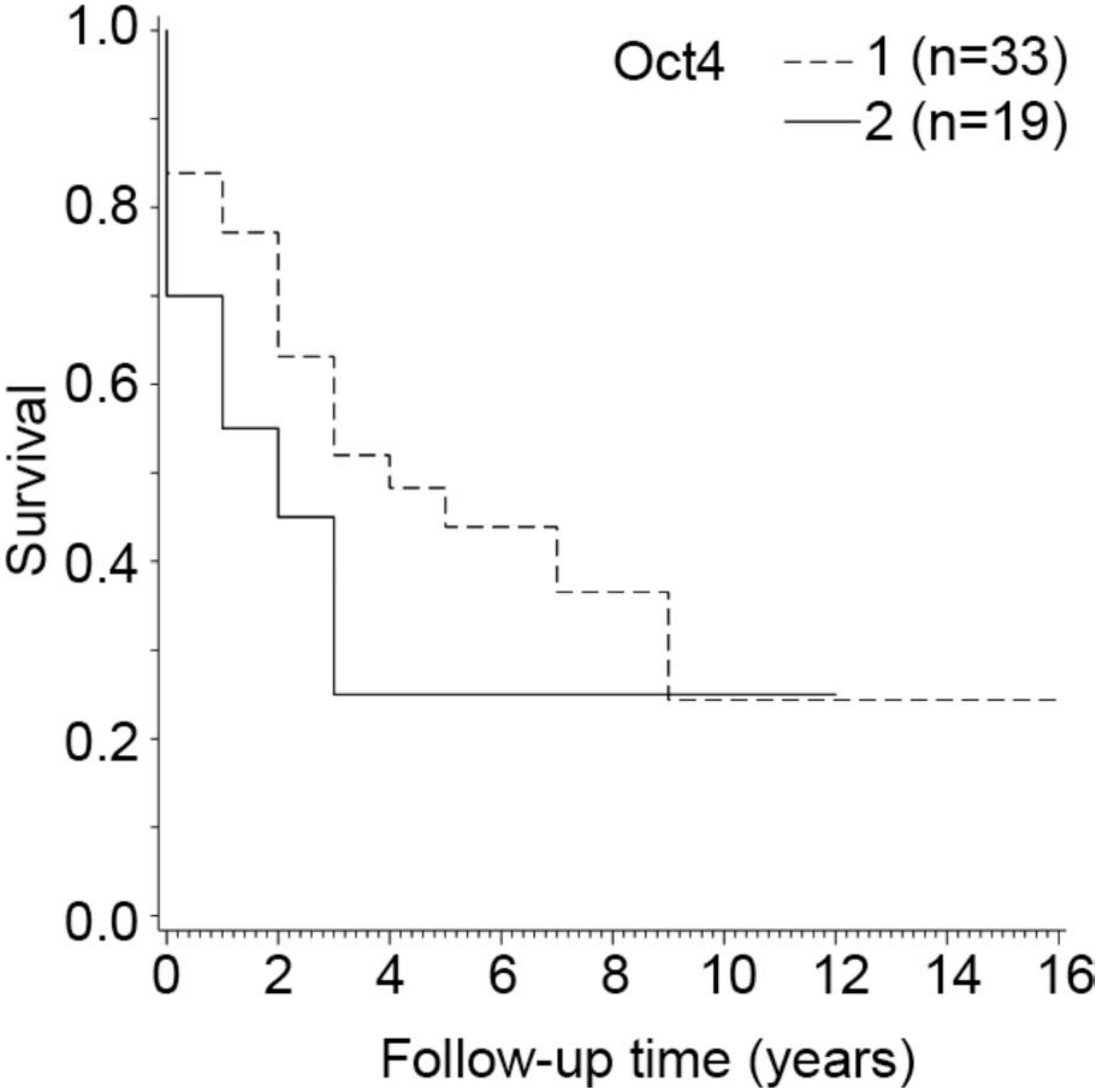

Supplementary Figure S4: The Kaplan–Meier 5 year overall survival curve from the HNSCC material (*n* = 52). The higher curve (1) represents the patients with a negative Oct4 expressing tumor, and the lower curve (2) represents HNSCC patients having strong Oct4 expression.

Supplementary Table S1. Primer design, accession numbers, annealing temperatures and amplicon lengths. Letters “m” and “h” in front of gene name refer to genes of mouse or human origin, respectively.

Gene	*Accession number*	*Annealing temperature*	*Primers*	*Product length*
mCD9	NM_007657	56ºC	5′-TGCAGTGCTTGCTATTGGAC-3′<br>5′-GGCGAATATCACCAAGAGGA-3′	219 bp
hCIP2A	NM_020890	57.5ºC	5′-GCTTGAAGTCCTTGCTCCTG-3′<br>5′-CAAGGCAACTCAAGCATTCA-3′	161 bp
mCIP2A	NM_172616	57ºC	5′-GCGCCATGTACTCAGTCAGA-3′<br>5′-AGGAAGCAGAAGGGTCACAA-3′	234 bp
hc-KIT	NM_000222	60ºC	5′-TCATGGTCGGATCACAAAGA-3′<br>5′-AGGGGCTGCTTCCTAAAGAG-3′	205 bp
mc-Kit	NM_021099	56ºC	5′-ATCCCGACTTTGTCAGATGG-3′<br>5′-AAGGCCAACCAGGAAAAGTT-3′	192 bp
hNANOG	NM_024865	57.5ºC	5′-GATTTGTGGGCCTGAAGAAA-3′<br>5′-AAGTGGGTTGTTTGCCTTTG-3′	154 bp
hOCT4	NM_002701	64ºC	5′-TGGAGAAGGAGAAGCTGGAGCAAAA-3′<br>5′-GGCAGATGGTCGTTTGGCTGAATA-3′	185 bp
mOct4	NM_013633	64ºC	5′-CACGAGTGGAAAGCAACTCA-3′<br>5′-AGATGGTGGTCTGGCTGAAC-3′	245 bp
mPLZF	NM_ 001033324	59ºC	5′-AACGGTTCCTGGACAGTTG-3′<br>5′-CCCACACAGCAGACAGAAGA-3′	172 bp
mPpia	NM_008907	63ºC	5′-CATCCTAAAGCATACAGGTCCTG-3′<br>5′-TCCATGGCTTCCACAATGTT-3′	164 bp
hRPL19	NM_000981	55ºC	5′-AGGCACATGGGCATAGGTAA-3′<br>5′-CCATGAGAATCCGCTTGTTT-3′	198 bp
mRPL19	NM_009078	55ºC	5′-GGACAGAGTCTTGATGATCTC-3′<br>5′-CTGAAGGTCAAAGGGAATGTG-3′	195 bp
mStra8	NM_009292	57ºC	5′-ATGCAATGTTGCTGAAGTGC-3′<br>5′-GGAAGCAGCCTTTCTCAATG-3′	161 bp

Supplementary Table S2. Used siRNA sequences

siRNA	5«-3«
CIP2A	CUGUGGUUGUGUUUGCACU(TT)
siOct4-1	GGAGAAGCUGGAGCAAAAC(TT)
siOct4-2	GCCGGGCUGGGUUGAUCCU(TT)
Nanog-2	TTGGTTTAAGTTCAAATGA(AT)
Nanog-4	CTGGAGGTCCTATTTCTCT(AA)
Sox2-4	TAGGACAGTTGCAAACGTG(AA)
Sox2-5	CAGCTCGCAGACCTACATG(AA)
